# Supplementary material for: Activity of Estrogen Receptor β Agonists in Therapy-Resistant Estrogen Receptor-Positive Breast Cancer
Source: Front Oncol. 2022 Apr 25;12:857590. doi: 10.3389/fonc.2022.857590 (PMC9097292; doi:10.3389/fonc.2022.857590)
Supplement: Supplementary file 1 [file DataSheet_1.pdf]

Supplemental Data

Preparation of MCSR-18-006

Synthetic Scheme for the Preparation of MCSR-18-006

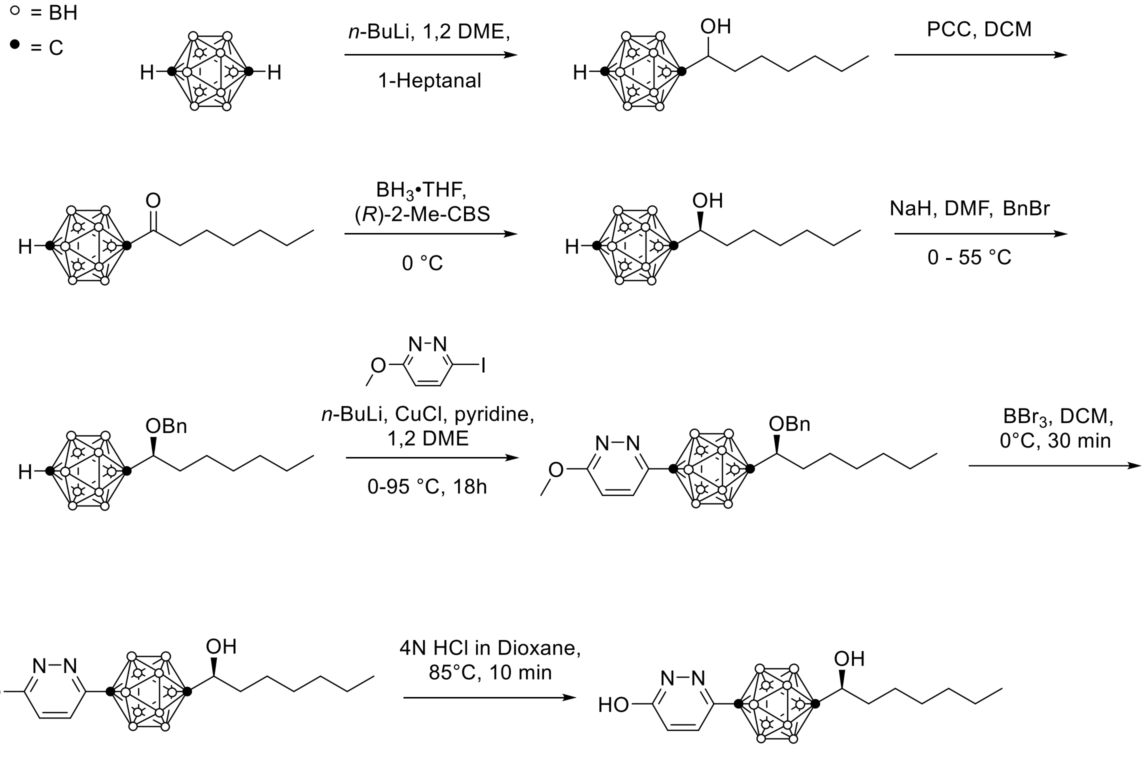

MCSR-18-006

Procedure for the synthesis of MCSR-18-006

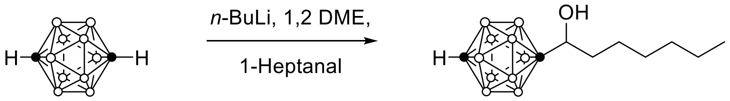

*1-(1-hydroxyheptyl)- 1,12-dicarba-closo-dodecaborane: n-*BuLi solution (2.5M in hexane, 4.4 ml) was added dropwise at 0 °C under Ar to a solution of 1,12-dicarba-*closo*-dodecaborane (1.44 g, 10 mmol) in 1,2 dimethoxyethane (50 ml) . We stirred the mixture at room temperature for 1 hour and then added 1-heptanal (1.55 ml, 11 mmol) at 0 ºC. This mixture was then stirred at room temperature overnight. We then poured the mixture into 1 M HCl aqueous solution (100 ml), and

performed extraction with ethyl acetate (3 X 25 ml). We then washed the combined organic phases with brine and dried them over $MgSO_4$. We evaporated the solvents and purified the residue using Teledyne Isco (RediSepRf column) to yield a colorless oil. Yield 1.4g. ^1H NMR ($CDCl_3$) δ 3.38-3.45(m, 1 H), 3.35-1.14 (m, 22H), 0.88 (t, 3 H), MS 258.291.

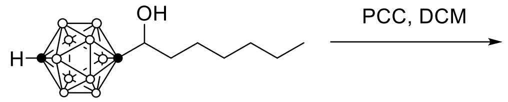

1-(Heptan-1-one)-1,12-dicarba-closo-dodecaborane: We suspended pyridinium chlorochromate (PCC, 1.7 g, 7.71 mmol) in anhydrous DCM (50 ml). We then added a solution of 1-(heptan-1-yl)-1,12-dicarba-*closo*-dodecaborane (1.3 g, 5.04 mmol) in DCM (10 ml), and stirred the reaction mixture at room temperature overnight. Diethyl ether (50 ml) was added to the mixture followed by molecular sieves, and then stirred for 1 h. We decanted the supernatant and washed the insoluble residue with dry ether (3 x 20 ml). We then passed the combined organic phases through a short column of Celite followed by evaporation. We then purified the residue with Teledyne Isco (RediSepRf column) to yield a colorless oil, yield 1.2g. ^1H NMR ($CDCl_3$) δ 1.16 (m, 21H), 0.88 (t, 3 H), MS 256.189.

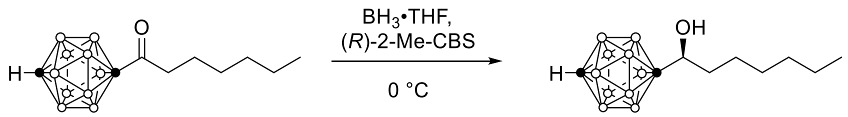

(R)-1-(1-hydroxyheptyl)- 1,12-dicarba-closo-dodecaborane: We added Borane-tetrahydrofuran complex (51 mL, 51 mmol, 1.0 M solution in THF, stabilized with 0.005 M N-isopropyl-N-methyl-tert-butylamine (NIMBA) followed by (R)-2-methyl-CBS- oxazaborolidine [(2-MeCBS] (5.1 mL, 5.1 mmol, 1.0 M solution in toluene) to 50 mL anhydrous THF. The reaction mixture was stirred at room temperature for 15 minutes. We then added 1-(Heptan-1-one)-1,12-dicarba-

closo-dodecaborane (1.3 g, 5.08 mmol) in 25 mL of anhydrous THF slowly over a period of 2 h at 0 °C. The reaction mixture was stirred overnight at room temperature. We then carefully quenched the mixture by the addition of 2.0 M HCl (80 mL) in small portions to control H_2 development. We added diethyl ether (100 mL) and washed the organic phase with brine and saturated $NaHC0_3$. We then dried the organic phase over $MgS0_4$, filtered, and evaporated. The residue was then purified by Teledyne Isco (RediSepRf column) to yield a colorless oil, yield 1.1g 81%. ^{1}H NMR ($CDCl_3$) δ 3.38-3.45(m, 1 H), 3.35-1.14 (m, 22H), 0.88 (t, 3 H), MS 258.291.

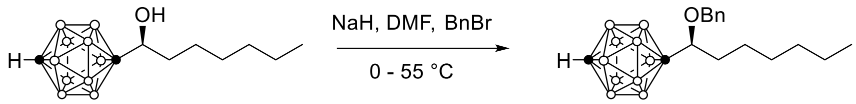

(R)-1-(1-benzyloxy)heptyl)- 1,12-dicarba-closo-dodecaborane: We added NaH (60% in mineral, 175 mg, 4.36 mmol) in one portion at 0 ºC to a solution of (*R*)-1-(1-hydroxyheptyl)- 1,12-dicarba-*closo*-dodecaborane (900 mg, 3.49 mmol) in anhydrous DMF (10 ml), , and then stirred at same temperature for 30 min. We then added BnBr (746 mg, 4.36 mmol) and stirred the reaction mixture at 55°C for 3 h. We then cooled it down to room temperature and added methanol (0.5 ml) slowly, followed by dilution with ethyl acetate (50ml), washing with water and brine and drying with Na_2SO_4. The solvents were then evaporated and the residue purified by Teledyne Isco (RediSepRf column) to yield a colorless oil, yield 1.1g 93%. ^{1}H NMR (300 MHz, $CDCl_3$) δ 7.28-7.35 (m, 5H), 4.63 (d, J = 11 Hz), 4.30 (d, J = 11 Hz), 3.12-3.23 (m, 1H), 2.75 (s, 1H), 0.98-1.40 (m, 10H), 0.96-3.30 (m, 10H), 0.87 (t, J = 7 Hz, 3H).

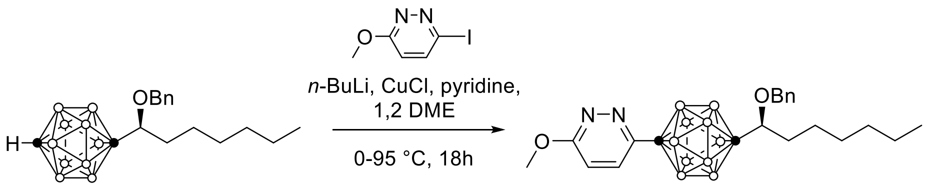

*(R)-1-(1-(benzyloxy)heptyl)-12-(6-methoxypyridazin-3-yl)-1,12-dicarba-closo-dodecaborane: n-*BuLi solution (2.5 M in hexanes, 274 µl, 0.68 mmol) at 0°C under Ar was added dropwise to a solution of (*R*)-1-(1-benzyloxy)heptyl)- 1,12-dicarba-*closo*-dodecaborane (197 mg, 0.56 mmol) in 1,2-dimethoxyethane (14.1 ml) . We then stirred the mixture at room temperature for 1h, and added CuCl (68 mg, 0.68 mmol) in one portion. Stirring was continued at room temperature for 1 h. We then added pyridine (637 µl, 7.91 mmol), and 3-iodo-6-methoxypyridazine (161 mg, 0.68 mmol) sequentially and heated the mixture at 95°C for 18 h. Following cooling, the reaction mixture was diluted with Et_2O and then stirred at room temperature for 3 h. We then filtered the insoluble materials through a short pad of silica, washed the filtrate with 10% aqueous $Na_2S_2O_3$ and brine, and dried the organic layer over Na_2SO_4, followed by filtration and concentration. We then purified the resulting residue using Combiflash (SiO_2, RediSepRf 12 g gold column, gradient: EtOAc/Hex) to yield 108 mg (42%) of a clear colorless oil. ^{1}H NMR (300 MHz, $CDCl_3$): δ 7.22-7.38 (m, 6H), 6.80 (d, *J* = 9.2 Hz, 1H), 4.65 (d, *J* = 11.0 Hz, 1H), 4.33 (d, *J* = 11.0 Hz, 1H), 4.09 (s, 3H), 3.23-3.29 (m, 1H), 1.41-3.66 (m, 10H), 1.01-1.44 (m, 10H), 0.85 (t, *J* = 7.0 Hz, 3H).

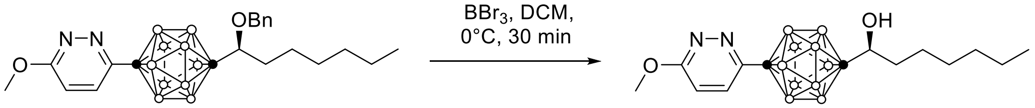

(R)-1-(1-hydroxyheptyl)-12-(6-methoxypyridazin-3-yl)-1,12-dicarba-closo-dodecaborane: 1 M solution of BBr_3 in CH_2Cl_2 (0.70 mL, 0.70 mmol) was added dropwise at 0 °C to a solution of *(R)*-1-(1-(benzyloxy)heptyl)-12-(6-methoxypyridazin-3-yl)-1,12-dicarba-*closo*-dodecaborane (91 mg, 0.20 mmol) in CH_2Cl_2 (2 ml). The mixture was stirred at 0 °C for 30 min and then poured into ice water and $NaHCO_3$. We then extracted the mixture 3x with CH_2Cl_2 (*note: 1-2% methanol was added to clarify the emulsion*). We washed the organic layer with brine, dried it over Na_2SO_4, and filtered and concentrated it *in vacuo*. We purified the resulting residue using Combiflash (SiO_2, RediSepRf 4g column, gradient: EtOAc/Hex) to yield 61 mg (83%) of a white powder. ^{1}H NMR (400 MHz, $CDCl_3$): δ 7.25 (d, *J* = 9.3 Hz, 1H), 6.80 (d, *J* = 9.3 Hz, 1H), 4.09 (s, 3H), 3.44-3.50 (m, 1H), 1.63-3.40 (m, 10H), 1.59 (d, *J* = 6.2 Hz, 1H), 1.11-1.49 (m, 10H), 0.87 (t, *J* = 6.9 Hz, 3H).

MCSR-18-006

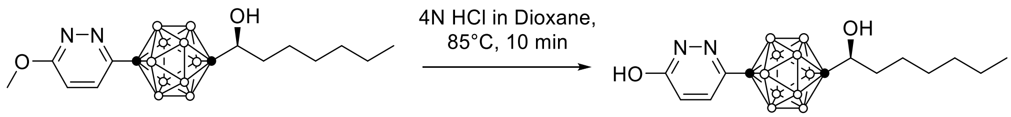

(R)-1-(1-hydroxyheptyl)-12-(6-hydroxypyridazin-3-yl)-1,12-dicarba-closo-dodecaborane : We added a solution of 4N HCl in Dioxane (0.5 mL) to *(R)*-1-(1-hydroxyheptyl)-12-(6-methoxypyridazin-3-yl)-1,12-dicarba-*closo*-dodecaborane (50 mg, 0.14 mmol). We then heated the mixture at 85 °C for 10 min, cooled it to room temperature, evaporated it to dryness with a gentle stream of argon, treated the residue with saturated aq. $NaHCO_3$ and extracted it 3x with ethyl acetate. We then washed the organic layer with brine, dried it over Na_2SO_4, filtered and concentrated it *in vacuo*. We purified the resulting residue using Combiflash (SiO_2, RediSepRf 4g gold column, gradient: MeOH/DCM) to yield 38 mg (79%) of a white powder. ^{1}H NMR (400 MHz, $CDCl_3$): δ 10.79 (br s, 1H), 7.14 (d, *J* = 10.0 Hz, 1H), 6.79 (d, *J* = 10.0 Hz, 1H), 3.40-3.50 (m, 1H), 1.66-3.30 (m, 10H), 1.62 (d, *J* = 6.3 Hz, 1H), 1.11-1.50 (m, 10H), 0.87 (t, *J* = 6.9 Hz, 3H). MS (APCI-): *m/z* calcd. for $C_{13}H_{27}B_{10}N_2O_2$ $(M-H)^-$ 351.3, found 351.1.

NMR Spectra for carborane compounds

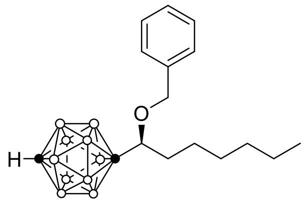

Molecular Weight: 348.53
Chemical Formula: $C_{16}H_{32}B_{10}O$

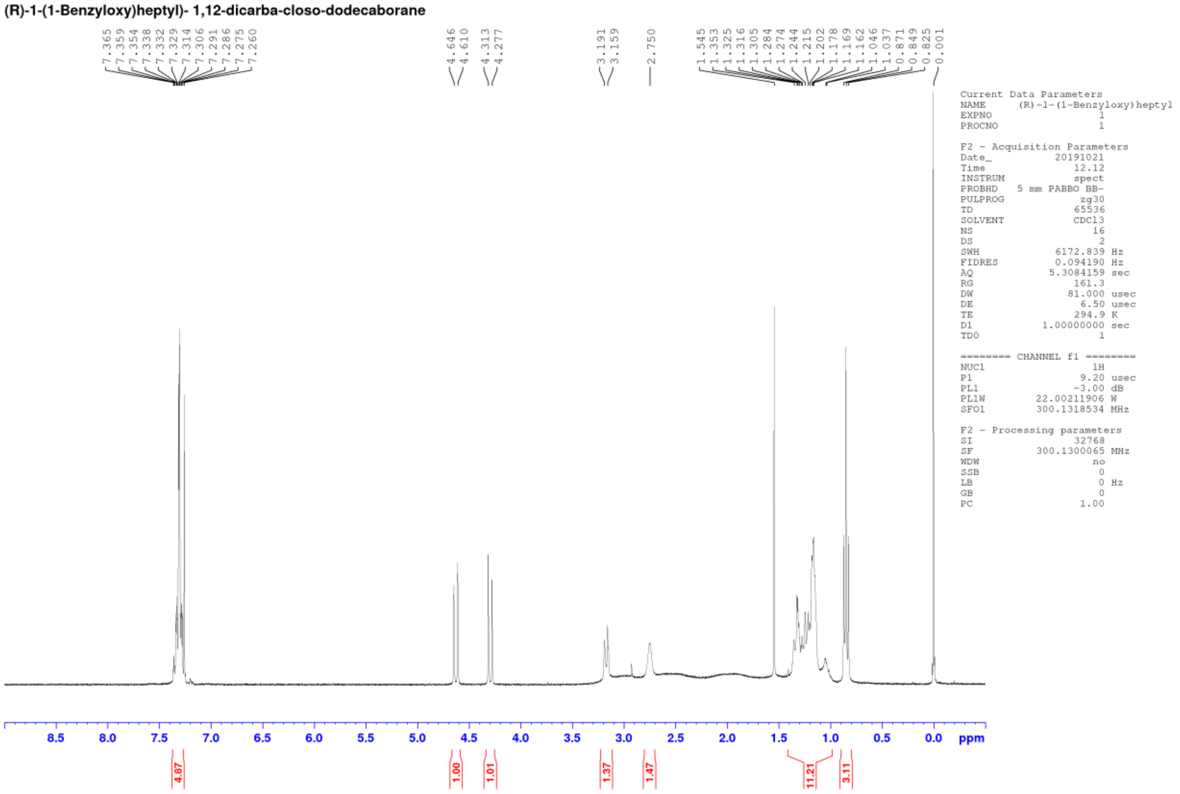

^{1}H NMR ($CDCl_3$, 300MHz) of Compound *(R)*-1-(1-benzyloxy)heptyl)- 1,12-dicarba-*closo*-dodecaborane.

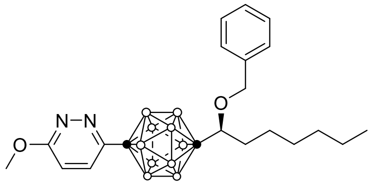

Molecular Weight: 456.63
Chemical Formula: $C_{21}H_{36}B_{10}N_2O_2$

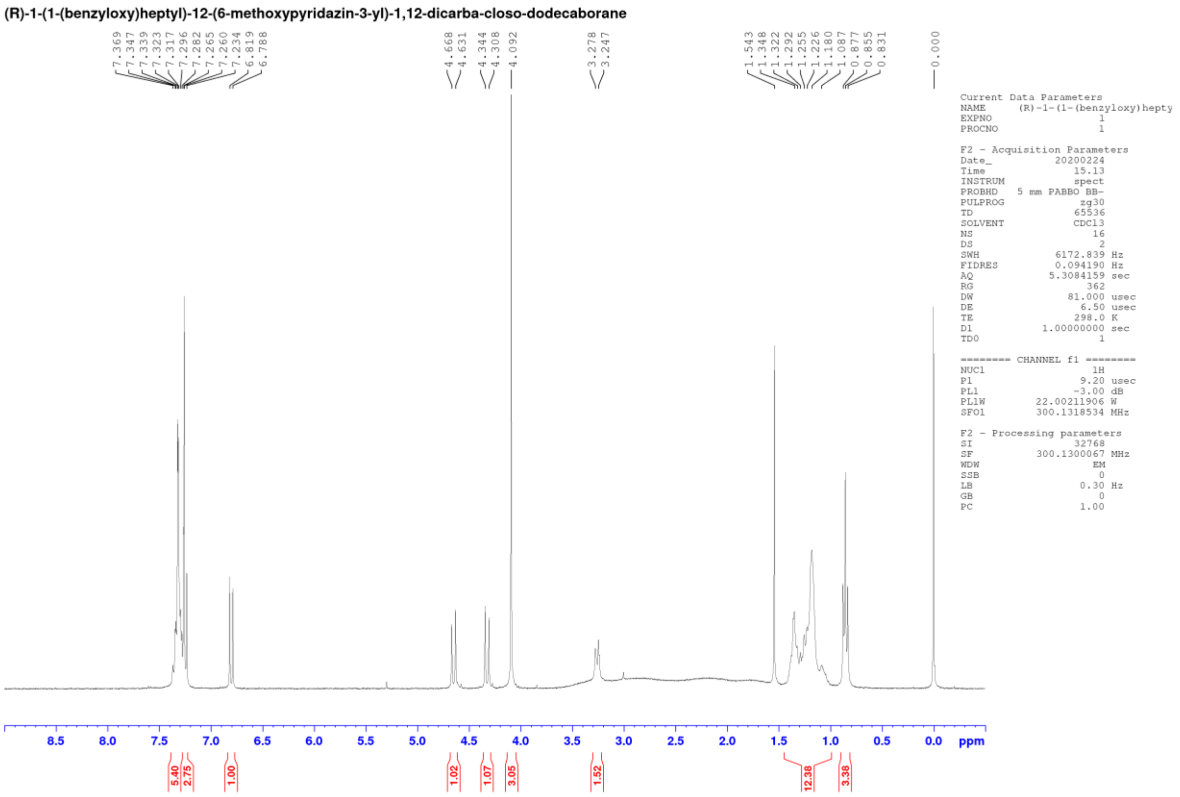

^{1}H NMR ($CDCl_3$, 300MHz) of Compound *(R)*-1-(1-(benzyloxy)heptyl)-12-(6-methoxypyridazin-3-yl)-1,12-dicarba-*closo*-dodecaborane.

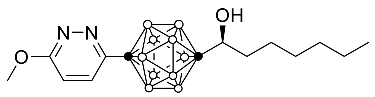

Molecular Weight: 366.51
Chemical Formula: $C_{14}H_{30}B_{10}N_2O_2$

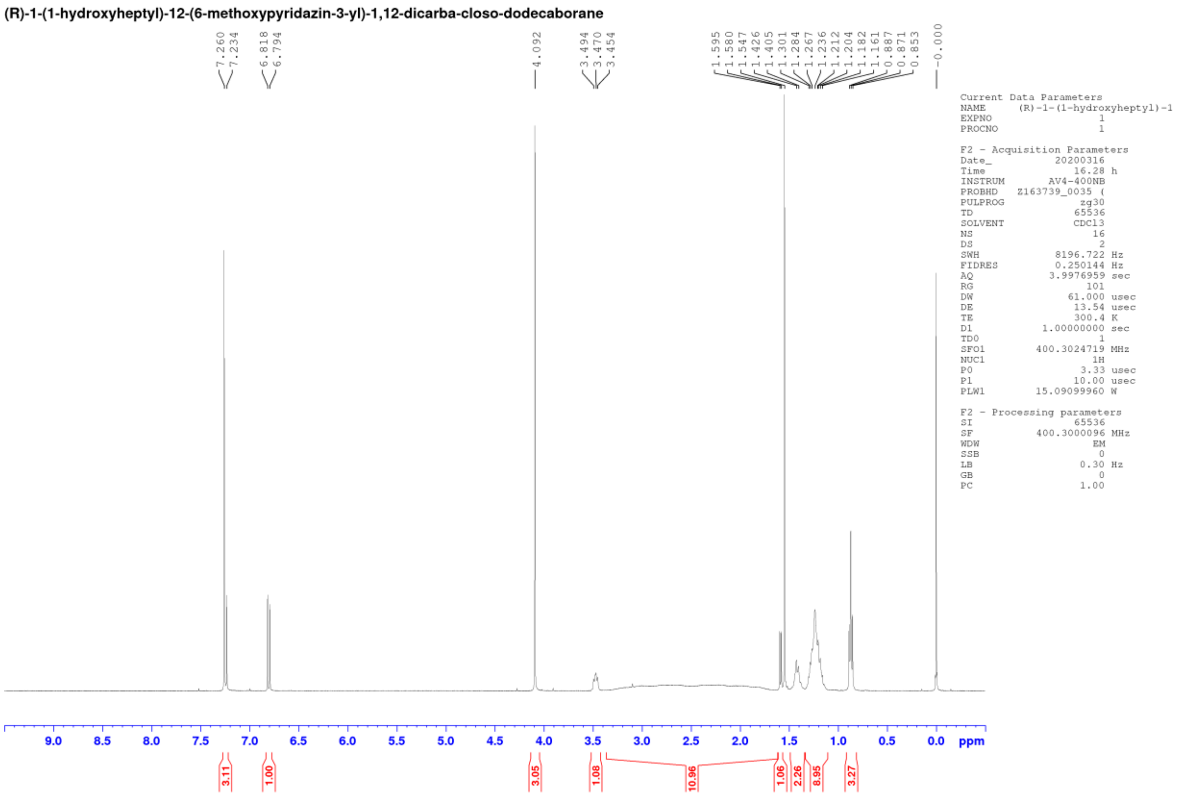

^{1}H NMR ($CDCl_3$, 400MHz) of Compound *(R)*-1-(1-hydroxyheptyl)-12-(6-methoxypyridazin-3-yl)-1,12-dicarba-*closo*-dodecaborane.

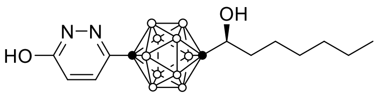

Molecular Weight: 352.48
Chemical Formula: $C_{13}H_{28}B_{10}N_2O_2$

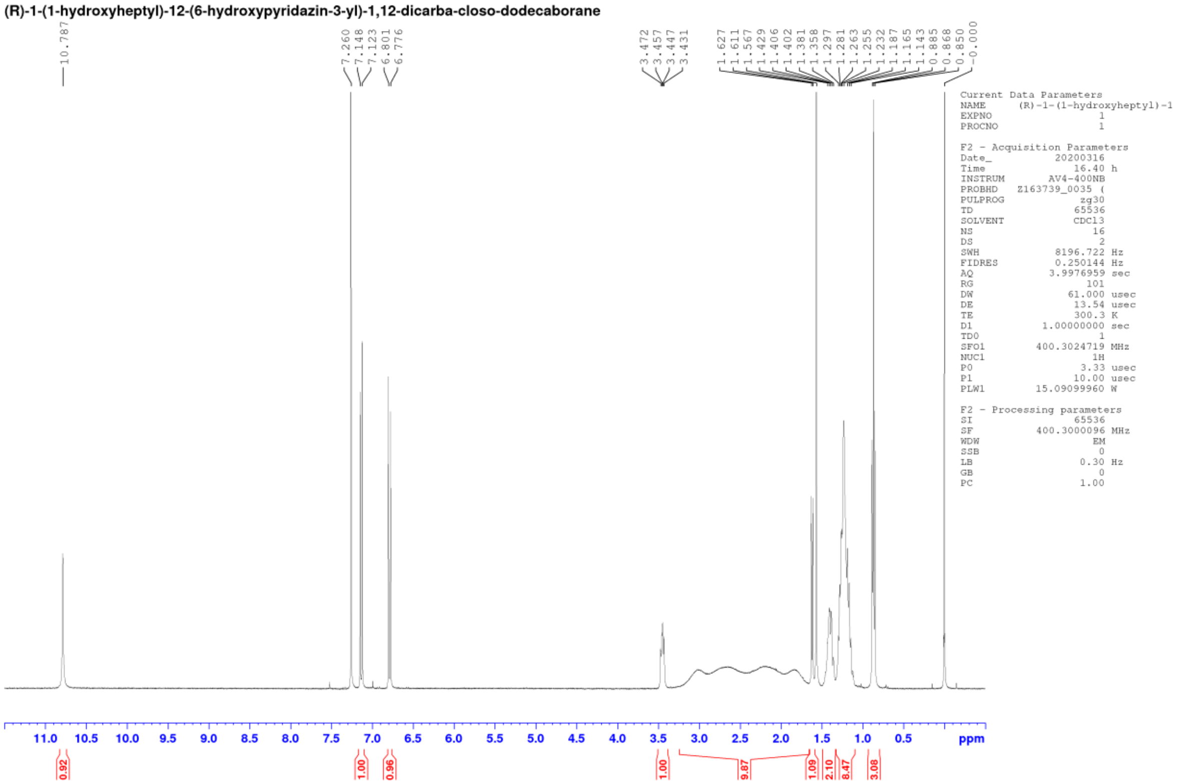

^{1}H NMR ($CDCl_3$, 400MHz) of Compound *(R)*-1-(1-hydroxyheptyl)-12-(6-hydroxypyridazin-3-yl)-1,12-dicarba-*closo*-dodecaborane (**MCSR-18-006**).

Supplemental Table 1. P-values and 95% confidence intervals (CIs) were determined for expression of the indicated genes in each cell line compared with that in the normal mammary epithelial line MCF10A.

qRT-PCR						
	**ESR2 Full Length**		**ESR2 All Isoforms**		**ESR1**	
**Cell Line**	**p-value**	**95% CI**	**p-value**	**95% CI**	**p-value**	**95% CI**
MCF10A		0.862-1.420		0.994-1.131		0.664-1.408
MCF7	0.7665	0.962-1.440	0.0524	2.999-3.998	0.0311	49770798-6034.474
MCF7-TamR	0.0035	3.240-4.291	0.0106	24.990-37.228	0.0179	9606.865-10728.515
MCF7-FasR	0.1196	1.502-1.526	0.0369	1.533-2.193	0.0227	429.422-493.742
MCF7-CDK6 O/E	0.1411	1.797-3.375	0.001	17.438-23.868	0.0147	2616.120-2864.450
MCF7-CDK4/6iR	0.0001	5.051-7.505	0.0018	31.568-54.566	0.0041	286.422-294.303
T47D	0.0265	6.536-8.202	0.001	72.211-81.421	0.0425	11597.865-15091.175
T47D-TamR	0.0009	4.698-5.294	0.0197	10.835-19.461	0.0203	3880.165-4398.077
T47D-FasR	0.0617	3.377-5.023	0.0194	26.819-46.711	0.0072	3071.065-3209.830
T47D-CDK4/6iR	0.0075	4.531-5.427	0.002	25.766-30.575	0.0331	7660.326-9398.086
ZR-75-1	0.0001	19.319-20.873	0.0145	201.031-219.808	0.052	230.515-318.321
MDA-MB-231	0.0404	3.616-4.985	0.0075	10.580-14.571	0.0496	1.577-1.896
MDA-MB-468	0.0003	5.041-5.283	0.0761	3.191-4.695	0.0158	76.036-84.139

Supplemental Table 2. 95% confidence intervals (CIs) and p-values were determined for ERE-Luciferase activity in ERα and ERβ overexpressing cells treated with the ERβ agonists OSU-ERb-12 and LY500307. Left & middle columns: p-values were determined for each protein compared with corresponding untreated control. Right column: p-values were determined for each concentration of agonist treated ERβ overexpressing cells compared with that of ERα overexpressing cells.

**ERE-Luciferase Activity**					
**OSU-ERb-12**					
	**ERα**		**ERβ**		**ERα vs ERβ**
**Concentration (µmol/L)**	**p-value**	**95% CI**	**p-value**	**95% CI**	**p-value**
0		0.92-1.08		0.83-1.17	1
0.01	0.1435	0.8296-0.9896	0.034	2.6241-4.2436	0.0232
0.03	0.0002	3.4941-3.7271	0.0053	35.7287-47.0914	0.0059
0.1	0.013	1.6786-1.9486	0.0012	37.5178-42.9054	0.0012
0.3	0.0215	1.1673-1.2809	0.009	34.5350-49.9702	0.0092
1	0.4306	0.9962-1.1483	0.0051	25.8500-33.8219	0.0048
5	0.0003	0.4797-0.6667	0.0014	18.9422-21.4334	0.0012
10	0.0263	0.3552-0.5607	0.0154	14.2808-22.4943	0.0129
**LY500307**					
	**ERα**		**ERβ**		**ERα vs ERβ**
**Concentration (µmol/L)**	**p-value**	**95% CI**	**p-value**	**95% CI**	**p-value**
0		0.92-1.08		0.83-1.17	1
0.01	0.0009	2.0095-2.2620	0.0037	73.7896-93.6231	0.0038
0.03	0.002	1.5881-1.7544	0.005	105.2260-107.7026	0.0051
0.1	0.0096	1.6203-1.9066	0.0102	63.9673-95.3686	0.0102
0.3	0.0029	1.8031-2.1447	0.0373	66.8043-80.2037	0.0372
1	0.0441	1.4546-2.0269	0.0019	67.8878-80.1651	0.0017
5	0.0221	1.4274-1.6103	0.0003	79.5217-79.9028	0.0014
10	0.046	1.1926-1.3828	0.0011	66.5517-77.7049	0.0011

Supplemental Table 3A. IC50 values, p-values , and 95% confidence intervals (CIs) were determined for cell viability in cytotoxicity assay with OSU-ERb-12 and LY500307. P-value (for IC50) for each cell line was determined by comparing with MCF10A.

Cytotoxicity						
	OSU-ERb-12			LY500307		
Cell Line	IC50 (µmol/L)	p-value	95% CI	IC50 (µmol/L)	p-value	95% CI
MCF10A	13.96		7.186-19.64	30.53		23.983-41.510
MCF7	11.22	0.0750	9.578-13.10	3.48	0.0225	3.396-3.444
MCF7-TamR	9.27	0.0249	4.563-14.68	7.01	0.0286	6.678-7.215
MCF7-FasR	4.86	0.0087	2.643-8.555	1.67	0.0200	1.449-1.917
MCF7-CDK6 OE	10.75	0.0538	9.313-12.31	7.66	0.0288	6.766-8.409
MCF7-CDK4/6iR	7.18	0.0089	5.391-9.214	6.89	0.0337	6.714-7.066
T47D	10.43	0.0403	9.715-11.29	7.29	0.0297	7.260-7.560
T47D-TamR	9.40	0.0301	9.439-9.461	6.66	0.0258	5.281-7.925
T47D-FasR	9.48	0.0304	9.457-9.483	7.75	0.0307	7.693-7.973
T47D-CDK4/6iR	9.08	0.0245	8.878-9.042	7.47	0.0281	6.230-7.838

Supplemental Table 3B. P-values were determined for cell viability in cytotoxicity assay with OSU-ERb-12 and LY500307. P-value (for IC50) for each cell resistant cell line was determined by comparing with corresponding parental cell line (MCF7 or T47D).

Cytotoxicity (parental vs drug resistant)		
	OSU-ERb-12	LY500307
Cell Line	p-value	p-value
MCF7		
MCF7-TamR	0.0118	0.0014
MCF7-FasR	0.0483	0.0043
MCF7-CDK6 OE	0.1302	0.0106
MCF7-CDK4/6iR	0.0031	0.0421
T47D		
T47D-TamR	0.0415	0.3096
T47D-FasR	0.0478	0.0915
T47D-CDK4/6iR	0.0271	0.4181

Supplemental Table 4. IC50 values, p-values , and 95% confidence intervals (CIs) were determined for viability of T47D cells in cytotoxicity assay with OSU-ERb-12, LY500307 and combination of drugs as indicated. P-value (for IC50) for each combination was determined by comparing with OSU-ERb-12 or LY500307 alone (as indicated).

**T47D Cytotoxicity**			
**Drug**	**IC50 (µmol/L)**	**p-value**	**95% CI**
OSU-ERb-12	14.10		14.035-14.271
OSU-ERb-12+ 0.5µmol/L 4OH-Tam	1.00	<0.0001	0.6828-1.53
OSU-ERb-12+ 0.25µmol/L Fas	4.47	0.0179	2.789-8.391
OSU-ERb-12+ 1µmol/L Elacestrant	6.18	<0.0001	5.450-7.024
OSU-ERb-12+ 0.75µmol/L MPP	3.29	0.0038	2.646-4.170

**T47D Cytotoxicity**			
**Drug**	**IC50 (µmol/L)**	**p-value**	**95% CI**
OSU-ERb-12	10.41		9.715-11.29
OSU-ERb-12+0.75µmol/L MPP	1.22	0.0005	0.8112-1.823
LY500307	7.29		7.131-7.708
LY500307+0.75µmol/L MPP	4.48	0.001	4.346-4.598

Supplemental Table 5. Cell viability, Bliss prediction, synergy ratio, synergy 95% CI, and p-values were determined at each drug combination (in T47D cells) as indicated.

**Bliss Independence Model**					
**Combination**	**Combination Viability**	**Bliss prediction**	**Synergy Ratio**	**Synergy 95% CI**	**p-value**
0.125 µmol/L OSU-ERb-12 + 0.025 µmol/L Tam	0.83	1.59	1.9	1.44-2.51	0.001
0.250 µmol/L OSU-ERb-12 +0.0 50 µmol/L Tam	0.83	1.54	1.84	1.42-2.39	0.001
0.500 µmol/L OSU-ERb-12 + 0.100 µmol/L Tam	0.86	1.8	2.1	1.66-2.65	<0.001
1.25 µmol/L OSU-ERb-12 + 0.250 µmol/L Tam	0.81	1.75	2.16	1.52-3.05	0.002
2.5 µmol/L OSU-ERb-12 + 0.500 µmol/L Tam	0.82	1.97	2.41	1.85-3.13	<0.001
5 µmol/L OSU-ERb-12 + 1 µmol/L Tam	0.54	1.27	2.35	1.83-3.02	<0.001

Supplemental Table 6. IC50 values, p-values , and 95% confidence intervals (CIs) were determined for cell viability in cytotoxicity assay (in T47D cells) with OSU-ERb-12 alone, MCSR-18-006 alone and combination as indicated. P-value (for IC50) for OSU-ERb-12 alone/combination were determined by comparing with MCSR-18-006 alone or respective combination treatment.

T47D Cytotoxicity			
Drug	IC50 (μmol/L)	p-value	95% CI
MCSR-18-006	33.68		30.82-36.64
OSU-ERb-12	10.41	0.0026	9.715-11.29

T47D Cytotoxicity			
Drug	IC50 (μmol/L)	p-value	95% CI
MCSR-18-006+0.5μmol/L 4-OH-Tam	39.23		36.56-41.01
OSU-ERb-12+0.5μmol/L 4-OH-Tam	1.02	<0.0001	0.6044-1.552

Supplemental Table 7. p-values and 95% confidence intervals (CIs) were determined for proliferation of MCF7 and T47D cells. P-value was determined by comparing with the percentage of cell proliferation in each drug treatment with that of the corresponding control (in each cell line).

Proliferation					
MCF7			T47D		
Treatment	p-value	95% CI	Treatment	p-value	95% CI
Control		50.125-53.475	Control		46.355-48.978
Fas 0.5μmol/L	0.002	18.996-23.504	Fas 0.5μmol/L	0.0033	26.163-28.237
OSU-ERb-12 0.5μmol/L	0.0096	36.073-51.861	OSU-ERb-12 0.5 μmol/L	0.0087	54.730-61.137
OSU-ERb-12 10μmol/L	0.0163	23.900-26.167	OSU-ERb-12 10 μmol/L	0.0074	12.722-20.678
Control		24.232-28.035	Control		46.355-48.978
Fas 0.5 μmol/L	0.001	12.180-13.687	Fas 0.5μmol/L	0.0189	26.796-33.871
LY500307 0.5 μmol/L	0.2491	39.082-41.918	LY500307 0.5μmol/L	0.02	57.681-62.986
LY500307- 3 μmol/L	0.0028	4.483-10.250	LY500307- 7μmol/L	0.0154	30.541-35.192

Supplemental Table 8. p-values and 95% confidence intervals (CIs) were determined for each phase of cell cycle in OSU-ERb-12- and LY500307-treated MCF7 and T47D cells. P-value for each phase for each treatment was determined by comparing with the percentage of cells present in the corresponding control (DMSO treated) cells.

Cell Cycle								
MCF7								
	Sub G0		G0/G1		S		G2/M	
Treatment	p-value	95% CI	p-value	95% CI	p-value	95% CI	p-value	95% CI
Control		-		66.396-67.338		13.971-15.896		15.313-17.354
OSU-ERb-12 0.5µmol/L	-	-	0.0200	55.239-61.028	0.0059	21.098-21.569	0.0890	16.706-21.561
OSU-ERb-12 11µmol/L	1.0000	0.087-0.313	0.0038	75.655-78.145	0.0347	9.219-11.581	0.0020	10.239-11.694
LY500307 0.5µmol/L	0.2254	0.187-0.413	0.0191	49.863-57.937	0.0493	19.719-24.414	0.0097	20.821-21.512
LY500307 3µmol/L	0.1221	0.338-1.195	0.4598	66.517-68.216	0.2556	14.843-16.090	0.2965	15.172-15.695
T47D								
	Sub G0		G0/G1		S		G2/M	
Treatment	p-value	95% CI	p-value	95% CI	p-value	95% CI	p-value	95% CI
Control		0.305-1.162		81.475-82.858		6.794-7.139		7.7664-9.803
OSU-ERb-12 0.5µmol/L	0.1942	1.084-4.382	0.0036	74.582-76.685	0.0015	11.931-12.402	1.0000	7.719-9.748
OSU-ERb-12 10µmol/L	0.2799	0.317-3.017	0.6010	84.704-82.096	0.0130	8.903-9.630	0.0749	6.199-7.867
LY500307 0.5µmol/L	0.2107	0.956-4.244	0.0018	73.166-75.834	0.0004	13.168-13.299	0.8758	7.779-9.954
LY500307 7µmol/L	0.0068	5.781-6.819	0.0079	42.248-53.019	0.0060	17.673-21.860	0.0135	15.508-17.092

Supplemental Table 9. p-values and 95% confidence intervals (CIs) were determined for induction of apoptosis in OSU-ERb-12- and LY500307-treated MCF7 and T47D cells. P-value (for apoptosis induction) for each treatment was determined by comparing with the percentage of apoptotic cells present in the corresponding control (DMSO treated) cells.

Apoptosis					
MCF7			T47D		
Treatment	p-value	95% CI	Treatment	p-value	95% CI
Control		4.56-6.64	Control		5.16-7.84
OSU-ERb-12 (0.5µmol/L)	0.4	6.03-7.97	OSU-ERb-12 (0.5µmol/L	0.3	4.77-7.29
OSU-ERb-12 (10µmol/L)	0.04	7.45-9.95	OSU-ERb-12 (10µmol/L)	0.0226	12-13.9
Control		4.56-6.64	Control		2.23-4.17
LY500307 (0.5µmol/L)	0.03	6.03-7.97	LY500307 (0.5µmol/L)	0.003	8.88-11.4
LY500307 (3µmol/L)	0.015	7.45-9.95	LY500307 (7µmol/L)	0.0005	9.26-13

Supplemental Table 10. p-values and 95% confidence intervals (CIs) were determined for assessing the colony forming ability in OSU-ERb-12- and LY500307-treated MCF7 and T47D cells. P-value (for percentage of colony formation) for each treatment was determined by comparing with the percentage of that in the corresponding control (DMSO treated) cells.

Colony Formation Assay				
	MCF7		T47D	
Treatment	p-value	95% CI	p-value	95% CI
Control		560.6272-622.0394		409.86-532.14
OSU-ERb-12 (3μmol/L)	0.050162	502.12-515.88	0.180841	533.9786-569.3546
OSU-ERb-12 (5μmol/L)	0.001899	315.0875-350.2457	0.011064	107.4305-227.2361
LY500307 (3μmol/L)	0.003087	96.5738-152.7594	0.014908	316.7503-437.9163
LY500307 (5μmol/L)	0.000701	0	0.005	21.04-24.93

Supplemental Table 11. p-values and 95% confidence intervals (CIs) were determined for assessing the cell migration ability in OSU-ERb-12- and LY500307-treated MCF7 cells. P-value (for percentage of cell migration) for each treatment was determined by comparing with the percentage of that in the corresponding control (DMSO treated) cells.

Cell Migration Assay		
	24 Hours	
Treatment	p-value	95% CI
Control		91.76-108.24
OSU-ERb-12 (5μmol/L)	0.0004368	61.0140-69.5791
OSU-ERb-12 (10μmol/L)	0.0026058	52.0192-62.1763
LY500307 (5μmol/L)	8.438E-06	23.4626-36.1402
LY500307 (10μmol/L)	2.756E-05	5.1938-11.0745

Supplemental Table 12: Patient demographics.

	Overall (n=37)
**Age at diagnosis**	
**Median [min, max]**	56 [35, 169]
**Race**	
**Asian**	1 (3%)
**Black or African American**	1 (3%)
**White**	35 (95%)
**Ethnicity**	
**NOT Hispanic or Latino**	37 (100%)
**BMI**	
**Median [min, max]**	28 [19, 47]
**Stage (pathologic)**	
**I**	5 (14%)
**II**	8 (22%)
**III**	15 (41%)
**IV**	7 (19%)
**Unknown**	2 (5%)
**Grade**	
**High grade (3)**	7 (19%)
**Intermediate grade (2)**	23 (62%)
**Low grade (1)**	4 (11%)
**Unknown**	3 (8%)
**ER status**	
**Positive**	37 (100%)
**PR status**	
**Negative**	4 (11%)
**Positive**	33 (89%)
**HER2 status**	
**Equivocal**	2 (5%)
**Negative**	34 (92%)
**Positive**	1 (3%)
**Chemotherapy**	
**No**	8 (22%)
**Yes**	29 (78%)
**Hormone therapy**	
**No**	1 (3%)
**Yes**	36 (97%)
**Targeted therapy**	
**No**	20 (54%)
**Yes**	17 (46%)

Figure S1

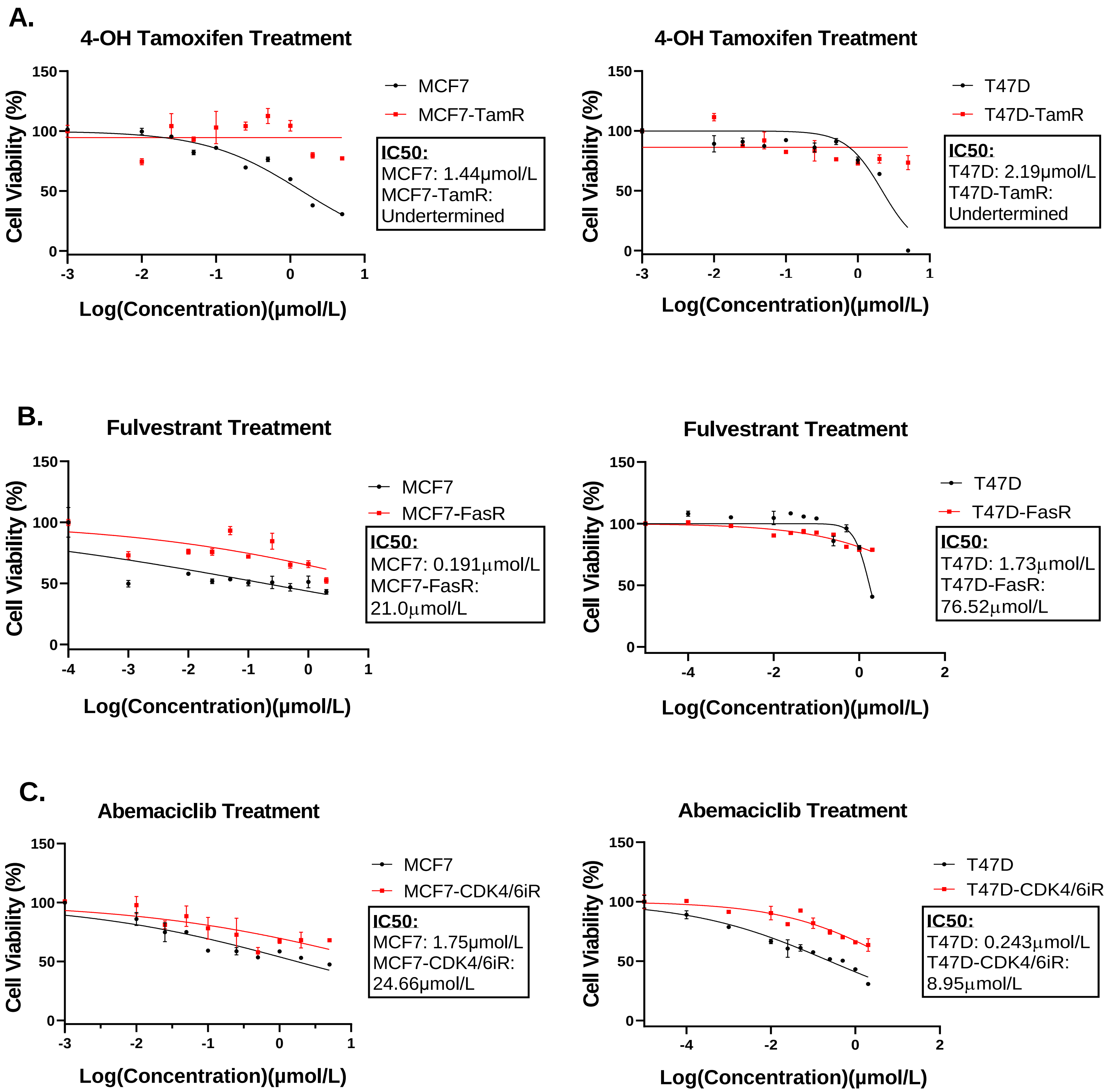

Supplemental Figure S1. Development of resistance in ERα+ breast cancer cells after chronic exposure to estrogen receptor antagonists and CDK4/6 inhibitor. Resistance was induced by chronic exposure of MCF7 and T47D cell lines to estrogen receptor antagonists and CDK4/6 inhibitor**.** Control cells were treated with DMSO in parallel. Control and resistant cells were treated for 72 hours with different concentrations of the drugs as indicated and the cell viability was measured. The percentage of viable cells relative to DMSO (control) are shown as mean ± SD. All the assays were performed in quadruplicate and repeated twice. Treatment with: **A,** 4-OH-tamoxifen **B,** fulvestrant **C,** abemaciclib.

Figure S2

OSU-ERb-12

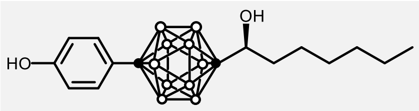

(*R*)-1-[1-(4-Hydroxyphenyl)-1,12-dicarba-*closo*-dodecaborane-12-yl]heptan-1-ol.

MCSR-18-006

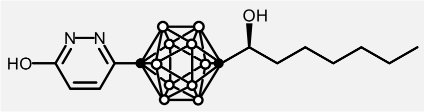

Black dots are carbon, white dots represent B-H

LY500307

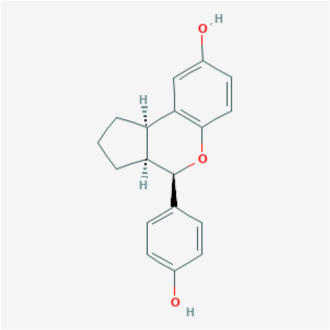

4-OH-Tamoxifen

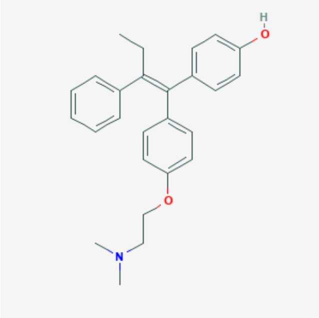

Elacestrant

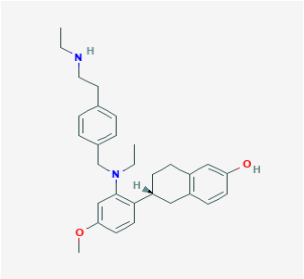

Fulvestrant

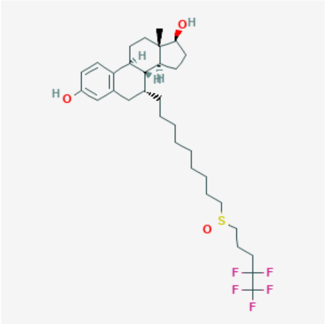

DPN (Diarylpropionitrile)

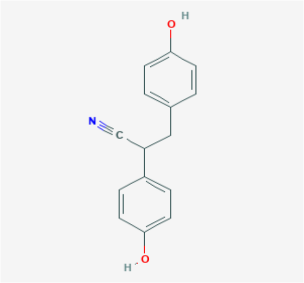

AC186

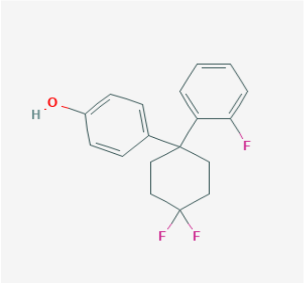

WAY 200070

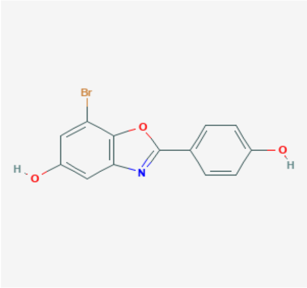

MPP (Methyl-piperidino-pyrazole)

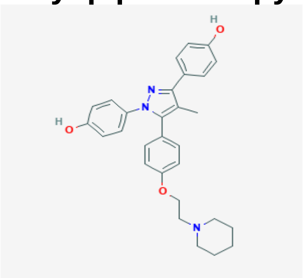

Supplemental Figure S2. Chemical Structures of the inhibitors used in the study

Figure S3

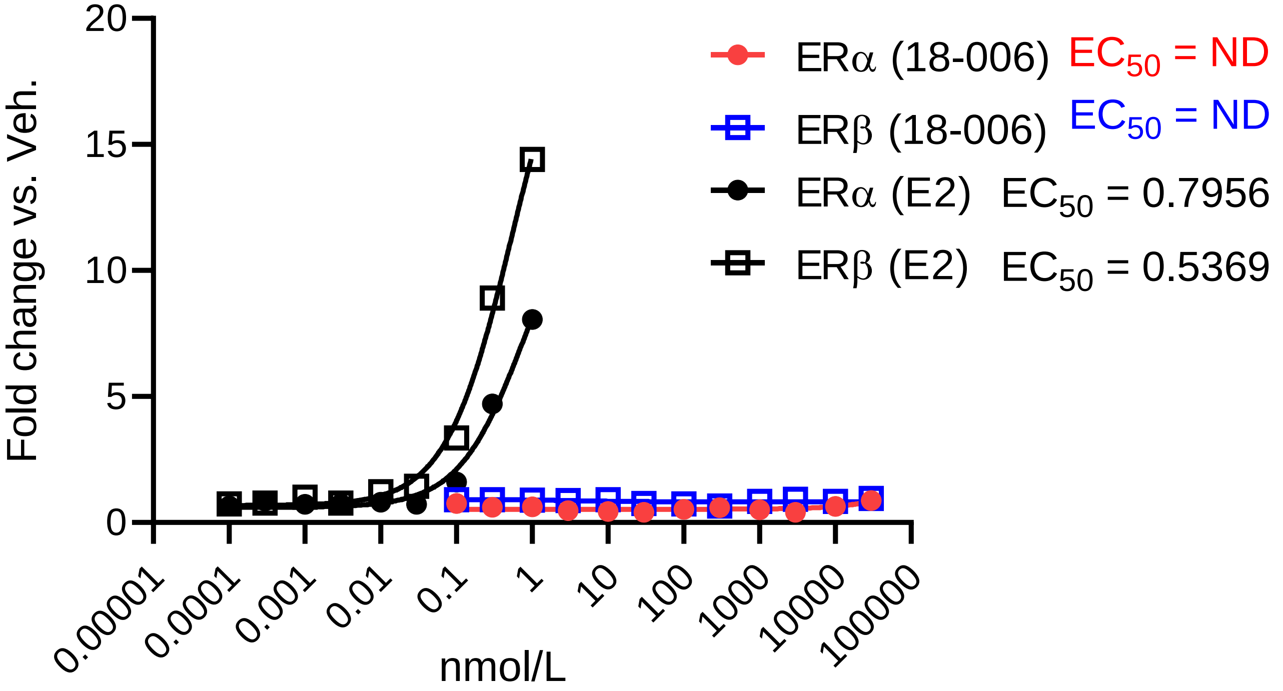

Supplemental Figure S3. ERα/ERβ driven ERE-Luciferase promoter activity is unaffected by MCSR-18-006, the chemical analog of the ERβ specific agonist OSU-ERb-12. The cell viability was measured in extracts prepared from human full-length ERα or ERβ expressing Sf9 cells in presence of vehicle or different concentrations of estrogen (E2) or MCSR-18-006 as indicated. Fold change in cell viability was determined by comparing with the vehicle control.

Figure S4

Supplemental Figure S4. Radio-ligand displacement assay demonstrated insignificant binding of the non-specific chemical analog MCSR-18-006, to ERα/ERβ. Recombinant human full-length ERα and ERβ were expressed in Sf9 cells, prepared the cell lysates, and the relative binding affinity of MCSR-18-006 was measured by radiolabeled ^{3}H estradiol competition binding assays. The IC50 the reference compound diethylstilbestrol was more than 100-fold less compared to MCSR-18-006 towards both ERα and ERβ. The binding affinities (Kis) of the reference compound for ERα and ERβ were 0.38 nmol/L and 0.22 nmol/L. The binding affinity value of MCSR-18-006 for ERα or ERβ could not be determined.

Radio-ligand Displacement Assay

DES (+) control

Reference Compound

Assay Name	Reference Compound	IC_{50}
Estrogen ERα	Diethylstilbestrol	1.32 nmol/L
Estrogen ERβ	Diethylstilbestrol	1.06 nmol/L

ERα: IC50 = 1.32 nmol/L, Ki = 0.38 nmol/L
ERβ: IC50 = 1.06 nmol/L, Ki = 0.22 nmol/L

Radio-ligand Displacement Assay

MCSR-18-006: Test Compound

Test Compound Experimental Results

Assay Name	Batch *	Spec.	Rep.	Conc.	% Inh.	IC_{50}*	K_i
Estrogen ERα	461679	hum	2	100 µmol/L	24	>100 µmol/L	
		hum	2	10 µmol/L	6		
		hum	2	1 µmol/L	3		
		hum	2	0.1 µmol/L	8		
		hum	2	10 nmol/L	-6		
		hum	2	1 nmol/L	3		
		hum	2	0.1 nmol/L	1		
		hum	2	10 pmol/L	4		
Estrogen ERβ	461680	hum	2	100 µmol/L	39	>100 µmol/L	
		hum	2	10 µmol/L	8		
		hum	2	1 µmol/L	-2		
		hum	2	0.1 µmol/L	0		
		hum	2	10 nmol/L	-7		
		hum	2	1 nmol/L	5		
		hum	2	0.1 nmol/L	4		
		hum	2	10 pmol/L	-3		

Figure S5

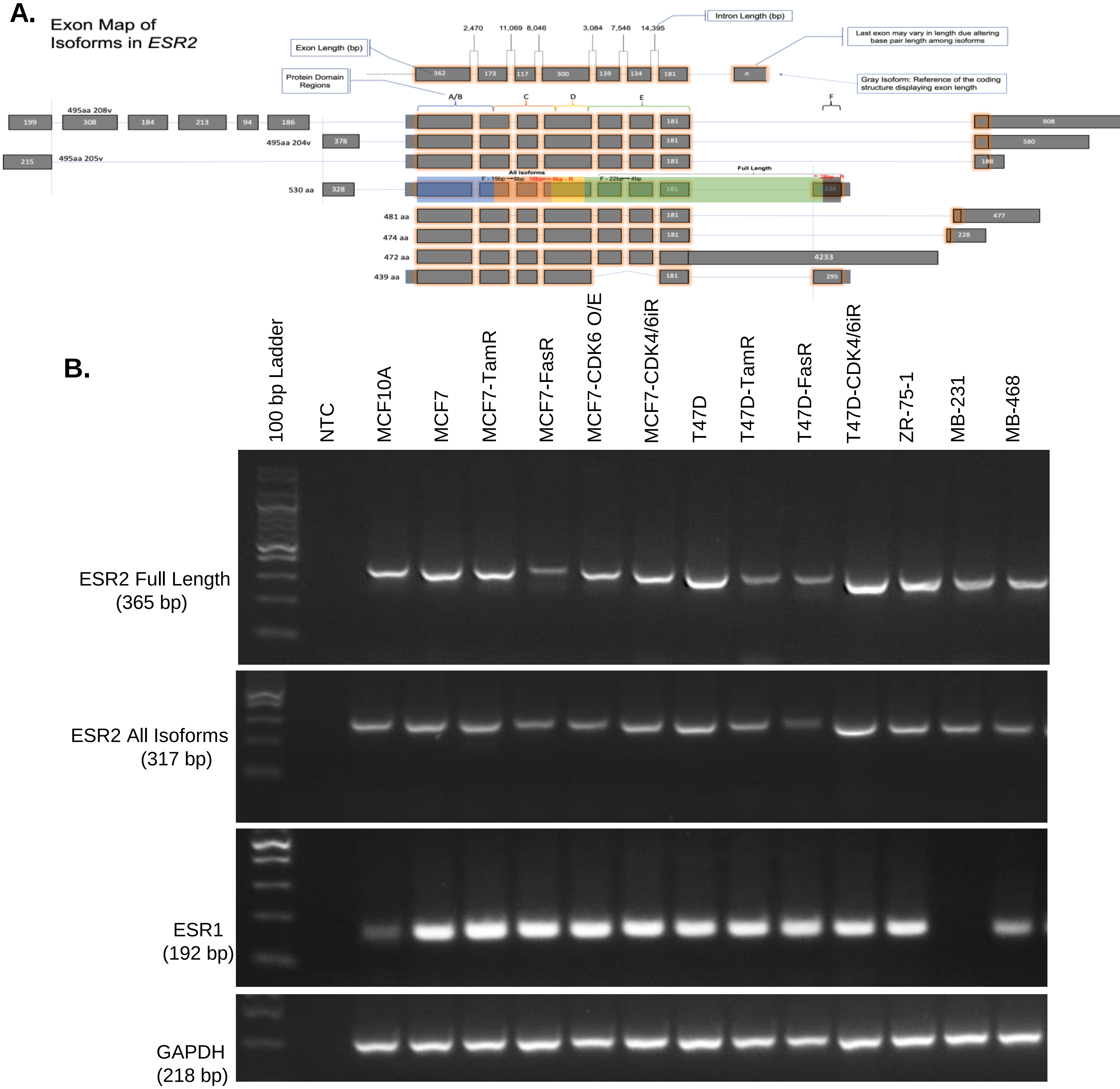

Supplemental Figure S5. A, Schematic diagram of primer design for qRT-PCR amplification of full length and all isoforms of *ESR2* gene. **B,** Agarose gel electrophoresis of qRT-PCR products of ESR2 full length, all isoforms, ESR1, and GAPDH showing a single band (of projected length) of corresponding gene product demonstrated specificity of the primers used in the study. qRT-PCR products of the indicated genes with the RNA isolated from the cell lines were separated on 2% agarose gels, stained with Gel Green dye, and photographed. Each assay was done in triplicate and repeated twice. Similar results were obtained. A representative picture of each gene product has been shown.

Figure S6

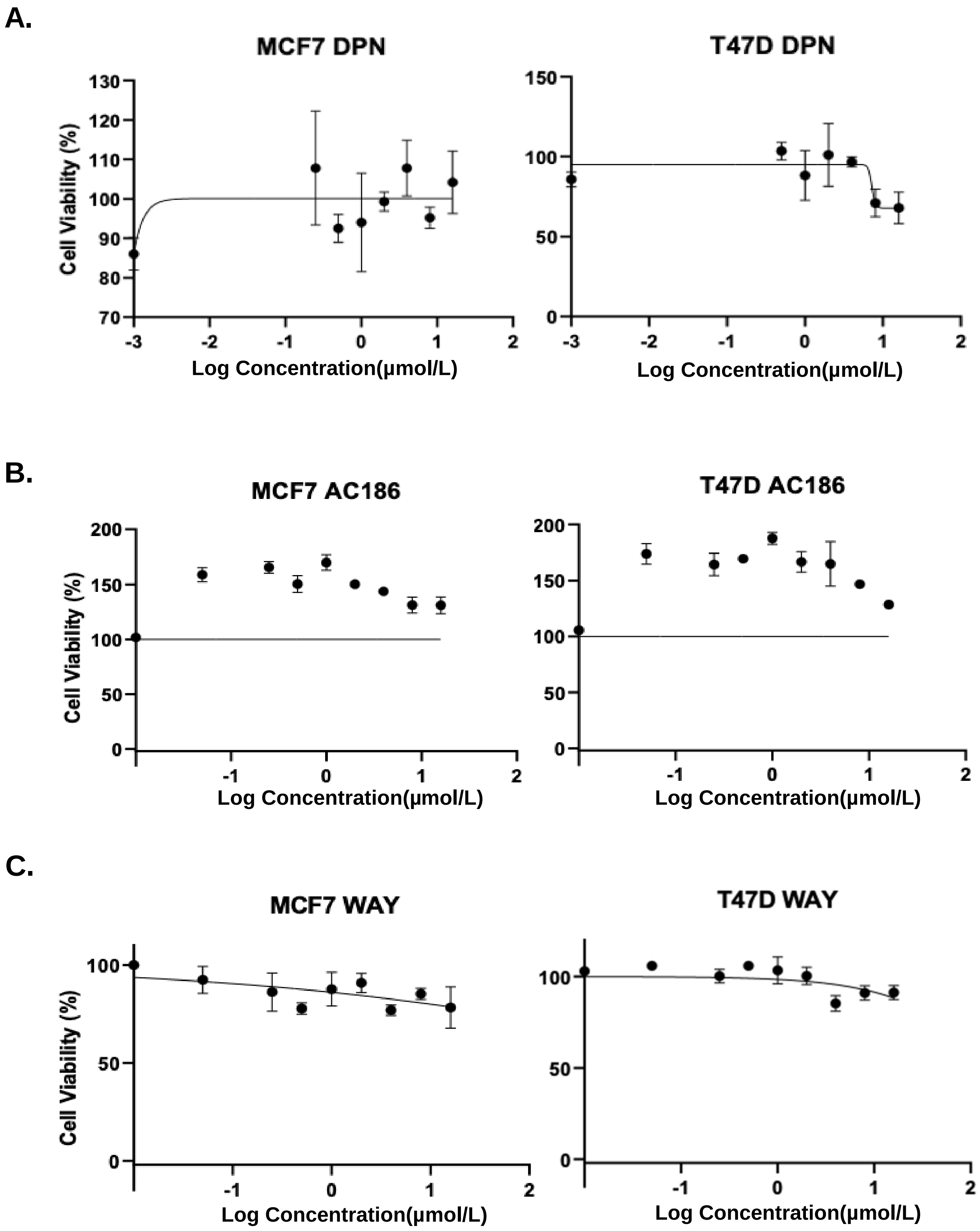

Supplemental Figure S6. Less selective ERβ agonists did not reveal cytotoxicity in ERα+ breast cancer cell lines. Cell viability assays were performed after 7 days of treatment of MCF7 and T47D cells with the agonists. Cell viability was determined relative to the viable cells upon treatment with the vehicle control (DMSO). Each assay was performed in quadruplicate and repeated twice. Data was presented as mean ± SD. Similar results were obtained. Representative data has been presented. Treatment with: **A,** DPN **B,** AC186 **C,** WAY200070.

Figure S7

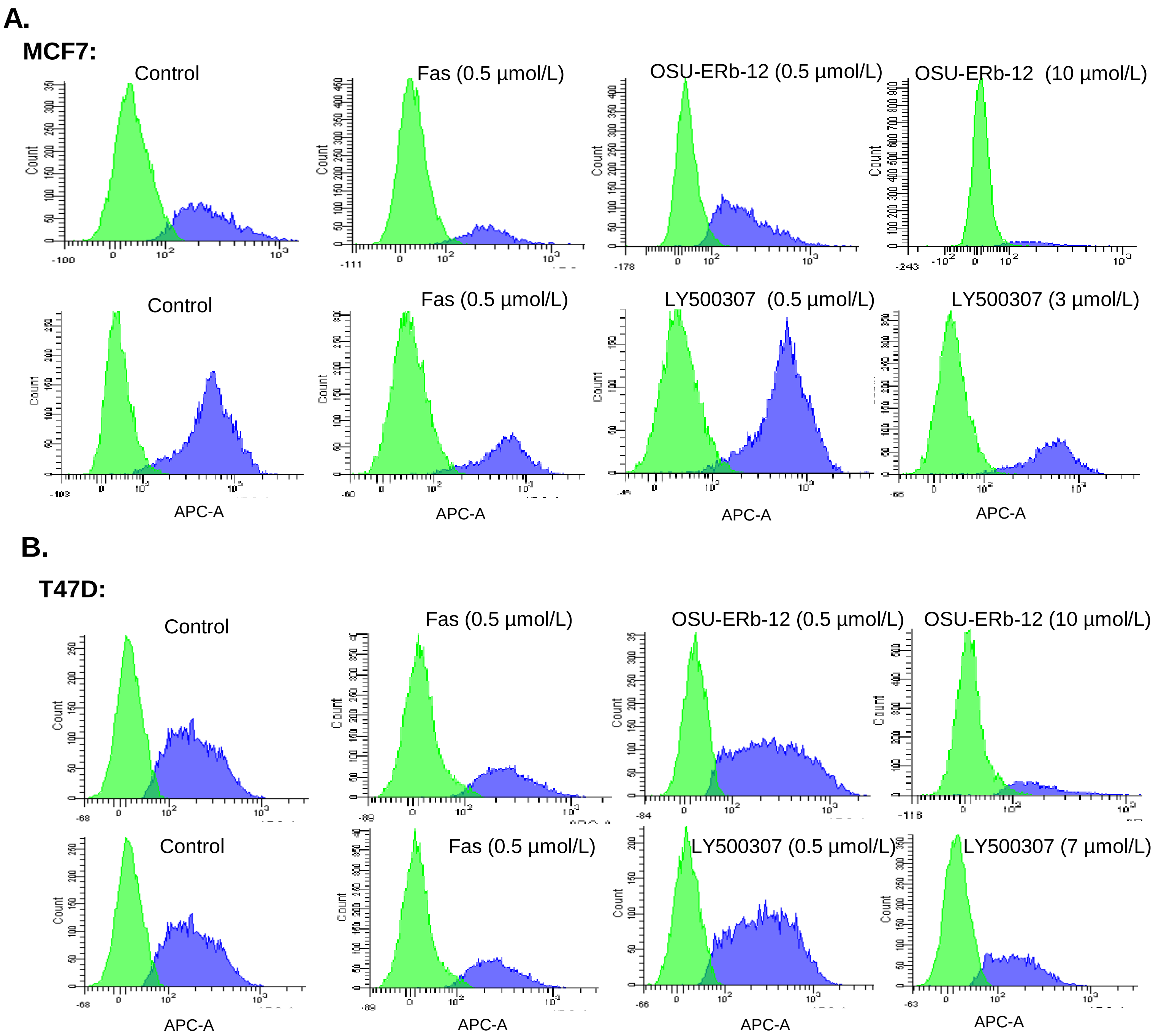

Supplemental Figure S7. Specific ERβ agonists OSU-ERb-12 and LY500307 inhibited proliferation of ERα+ breast cancer cells. Cell proliferation was measured after treatment with DMSO (control), fulvestrant, OSU-ERb-12 or LY500307 for 3 days. Washed cells were processed and proliferation was measured by FACS analysis as described in detail before (Materials and Methods). Each experiment was done in triplicate and repeated twice. Similar results were obtained. A representative profile has been presented. **A,** MCF7 **B,** T47D.

Figure S8

Cell Cycle Analysis

A.

MCF7:

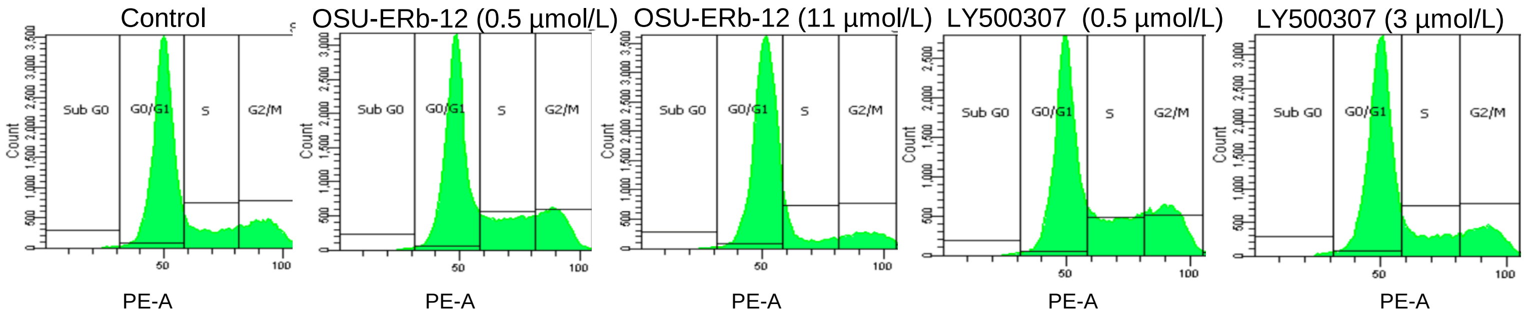

B.

T47D:

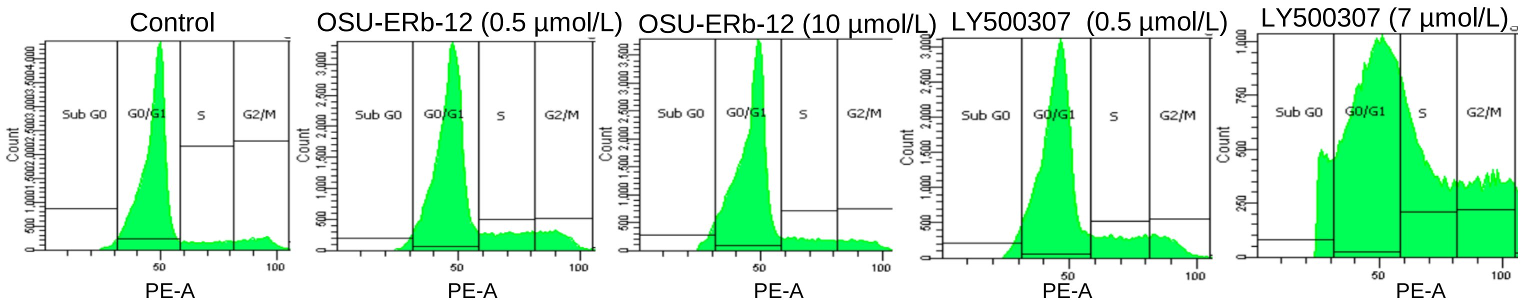

Supplemental Figure S8. Specific ERβ agonists OSU-ERb-12 and LY500307 caused S and/or G2/M phase arrest in ERα+ breast cancer cells. Cell cycle analyses were performed after treatment of cells with DMSO (control), OSU-ERb-12 or LY500307 for 3 days. Washed cells were processed and cell cycle profile was measured by FACS analysis as described in detail before (Materials and Methods). Each experiment was done in triplicate and repeated twice. Similar results were obtained. A representative profile has been presented. **A,** MCF7 **B,** T47D.

Figure S9

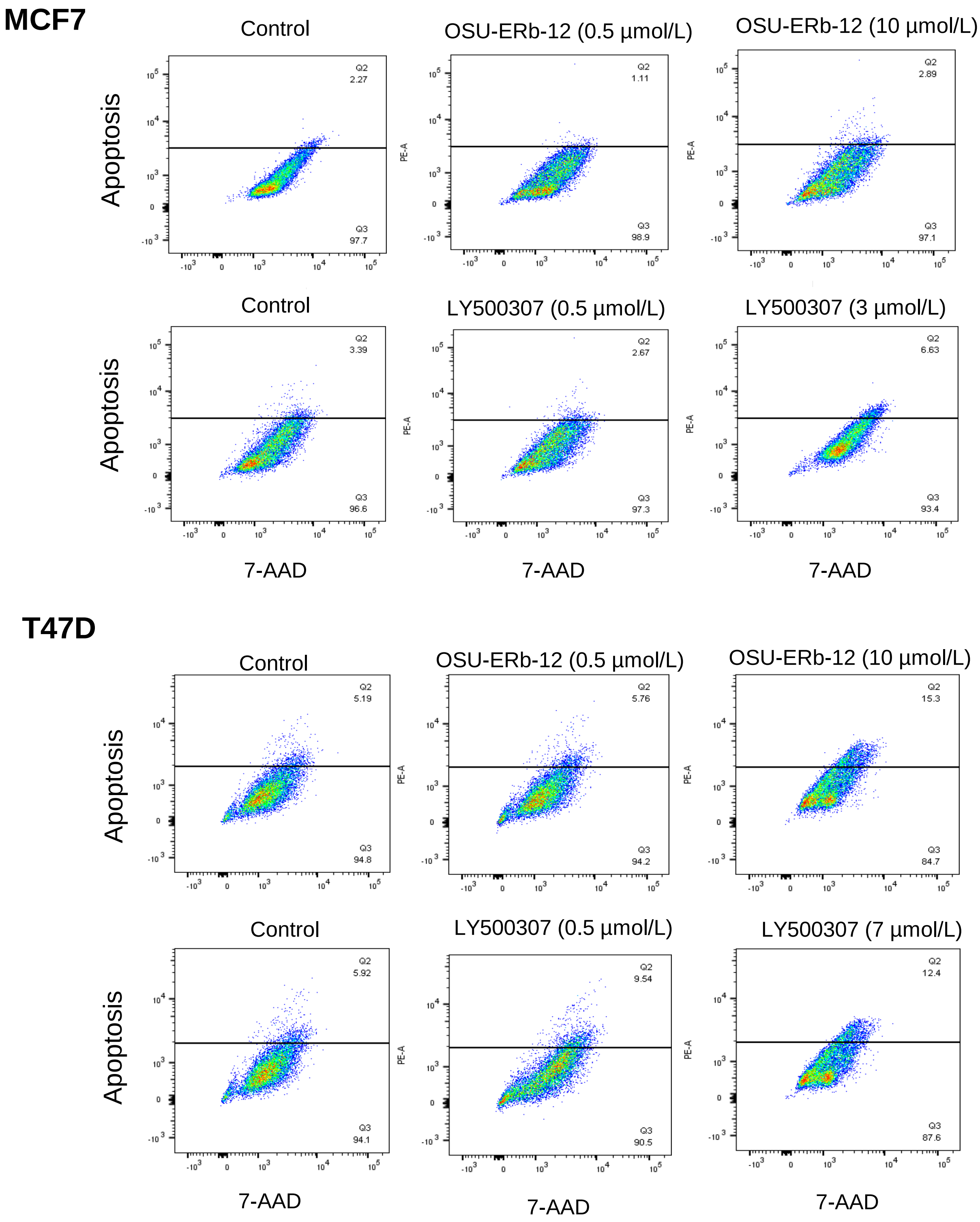

Supplemental Figure S9. Specific ERβ agonists OSU-ERb-12 and LY500307 induced apoptosis in ERα+ breast cancer cells. Apoptosis was measured after treatment of cells with DMSO (control), OSU-ERb-12 or LY500307 for 48 hours. Washed cells were processed, stained and apoptosis was measured by FACS analysis as described in detail before (Materials and Methods). Each experiment was done in triplicate and repeated twice. Similar results were obtained. A representative profile has been presented. **Top,** MCF7 **Bottom,** T47D.
